# Supplementary material for: Light Alcohol Consumption Promotes Early Neurogenesis Following Ischemic Stroke in Adult C57BL/6J Mice
Source: Biomedicines. 2023 Apr 2;11(4):1074. doi: 10.3390/biomedicines11041074 (PMC10136277; doi:10.3390/biomedicines11041074)
Supplement: Supplementary file 1 [file biomedicines-11-01074-s001.zip › biomedicines-2280282-supplementary.pdf]

Supplemental Figures

Figure S1

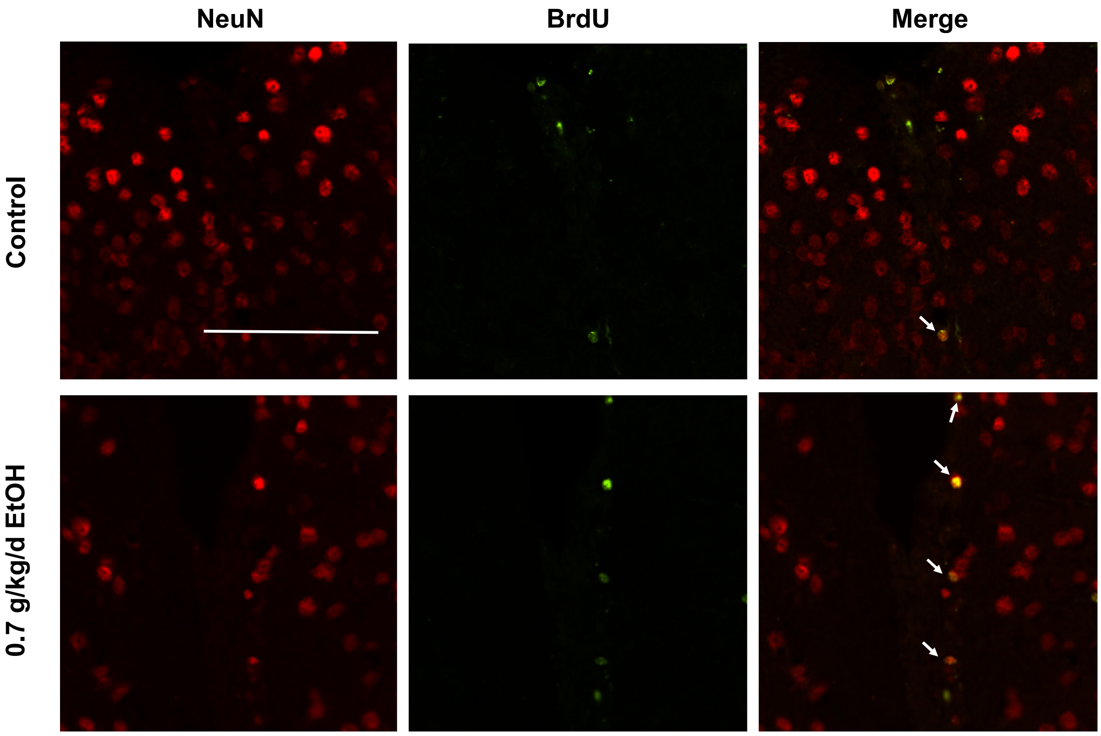

Figure S1. Effect of LAC on baseline neurogenesis in the SVZ. Representative double staining of BrdU and NeuN at 60x magnification. Scale bar = 100 µm.

Figure S2

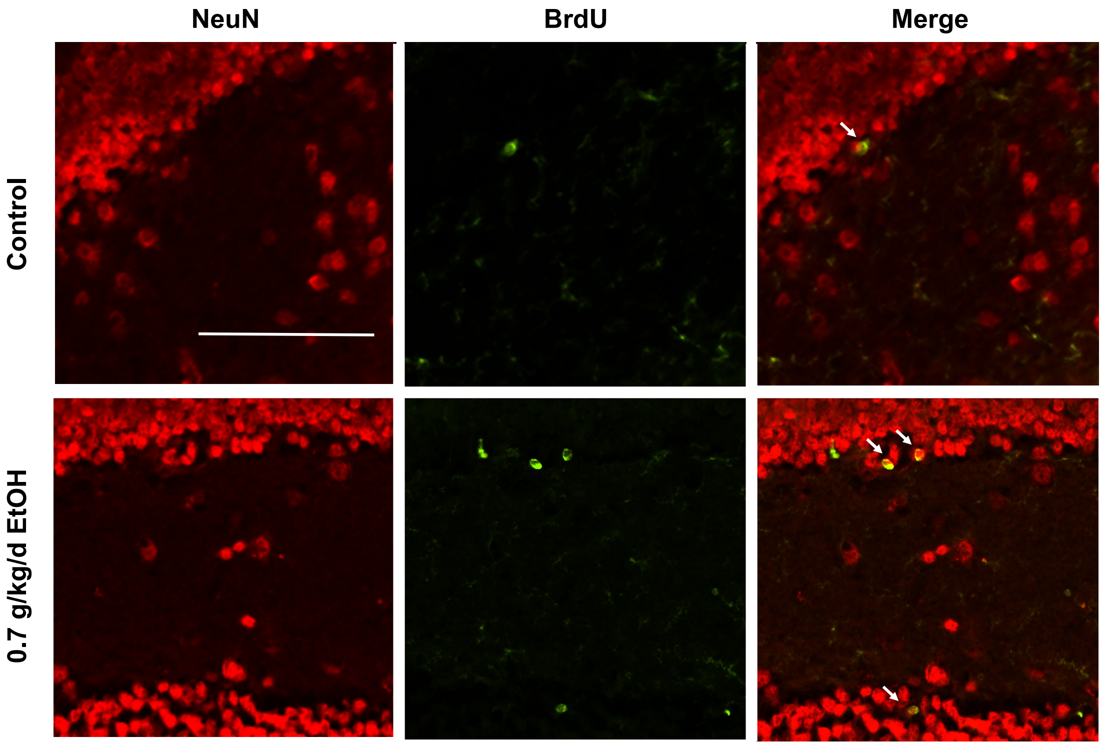

Figure S2. Effect of LAC on baseline neurogenesis in the DG. Representative double staining of BrdU and NeuN at 60x magnification. Scale bar = 100 μm.

Figure S3

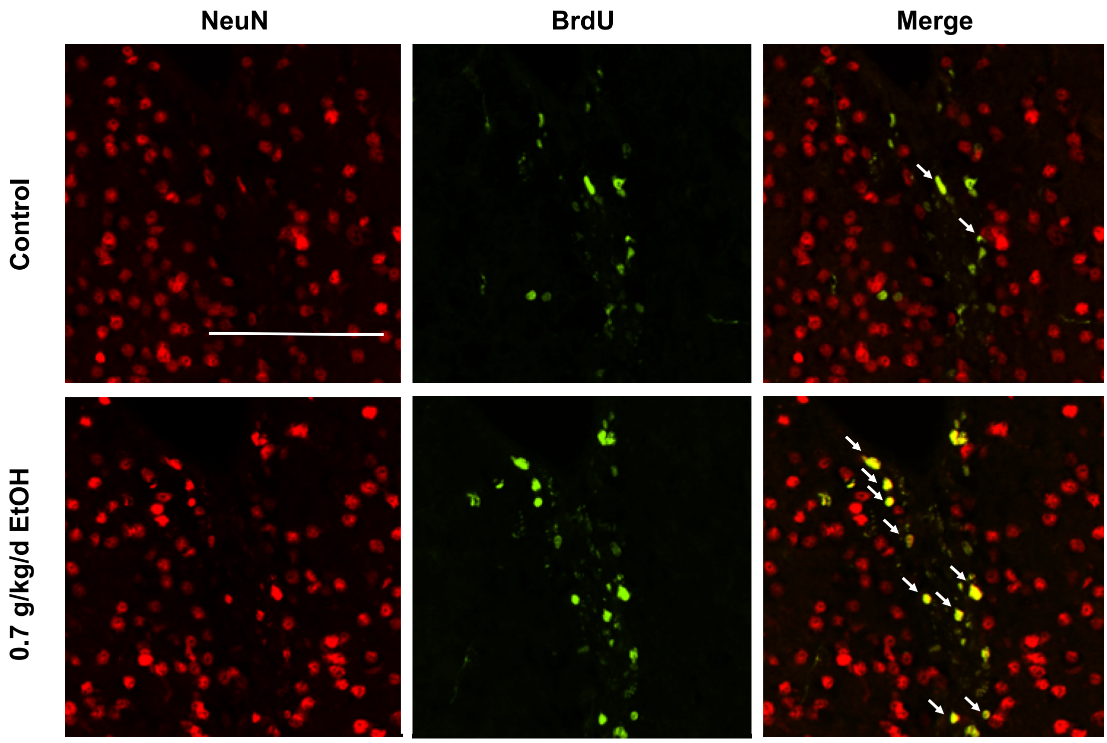

Figure S3. Effect of LAC on post-ischemic neurogenesis in the SVZ. Representative double staining of BrdU and NeuN at 60x magnification. Scale bar = 100 μm.

Figure S4

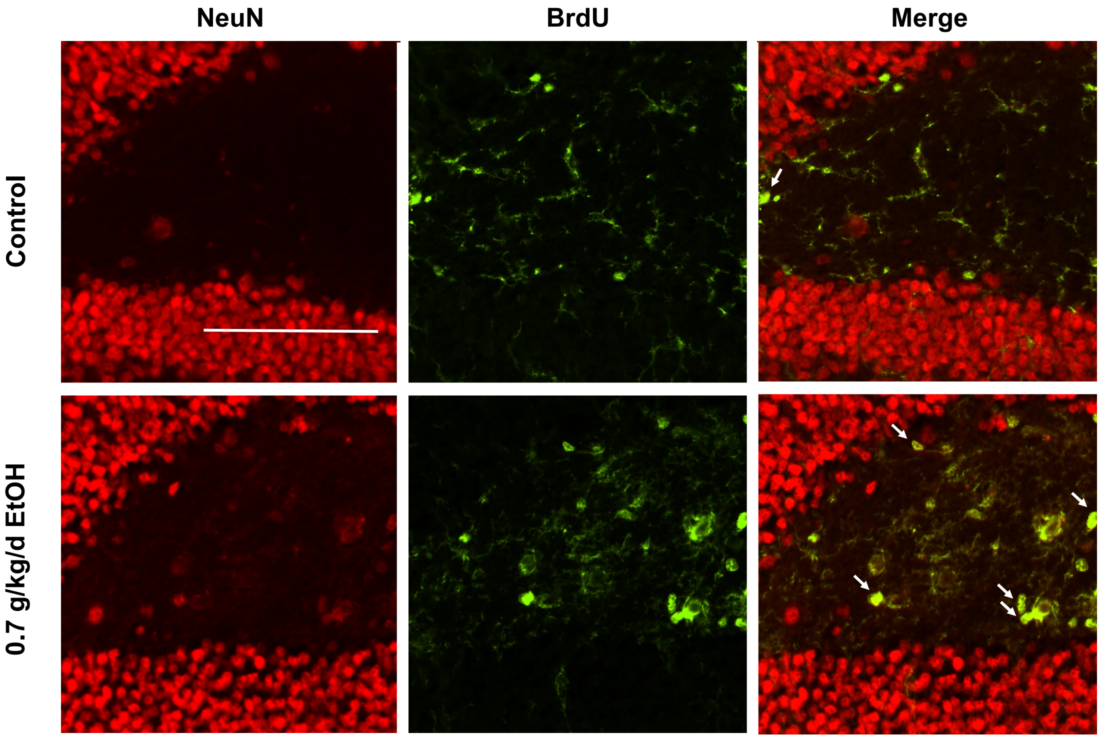

Figure S4. Effect of LAC on post-ischemic neurogenesis in the DG. Representative double staining of BrdU and NeuN at 60x magnification. Scale bar = 100 μm.
